# Supplementary material for: Spatial overlap of sea ice-associated predators and prey in western Hudson Bay
Source: PLoS One. 2026 Feb 2;21(2):e0328953. doi: 10.1371/journal.pone.0328953 (PMC12863486; doi:10.1371/journal.pone.0328953)
Supplement: S1 Table — (DOCX) [file pone.0328953.s001.docx]

**S1 Table.** Flight distance in km and duration in minutes per ordinal day per year (days in grey are non-survey flights and were not included in the sample effort).

| **Year** | **Julian day** | **Distance (Km)** | **Time (min.)** |
| --- | --- | --- | --- |
| **2019** | **110** | **35.8** | **14** |
| **2019** | **111** | **707.1** | **505** |
| **2019** | **112** | **646.2** | **472** |
| **2019** | **113** | **59.8** | **36** |
| **2019** | **114** | **4.1** | **25** |
| **2019** | **115** | **326.7** | **167** |
| **2019** | **116** | **661.9** | **389** |
| **2019** | **117** | **0.9** | **6** |
| **2019** | **118** | **660.0** | **421** |
| **2019** | **119** | **498.0** | **382** |
| **2019** | **120** | **126.4** | **122** |
| **2022** | **111** | **0.4** | **4** |
| **2022** | **112** | **740.0** | **448** |
| **2022** | **113** | **4.0** | **35** |
| **2022** | **114** | **136.3** | **98** |
| **2022** | **115** | **597.9** | **372** |
| **2022** | **116** | **952.1** | **507** |
| **2022** | **117** | **951.3** | **466** |
| **2022** | **118** | **715.3** | **429** |
| **2022** | **119** | **325.2** | **163** |
| **2022** | **124** | **530.4** | **268** |
| **2022** | **125** | **369.8** | **206** |
| **2023** | **107** | **255.9** | **138** |
| **2023** | **108** | **702.2** | **497** |
| **2023** | **109** | **1048.3** | **444** |
| **2023** | **110** | **481.9** | **252** |
| **2023** | **111** | **0.6** | **5** |
| **2023** | **115** | **586.3** | **274** |
| **2023** | **118** | **217.5** | **126** |
| **2023** | **119** | **923.2** | **447** |
| **2023** | **120** | **2.9** | **43** |
| **2023** | **121** | **279.4** | **205** |
| **2023** | **122** | **3.3** | **43** |
| **2023** | **123** | **301.9** | **234** |
| **2023** | **124** | **0.4** | **3** |
| **2024** | **107** | **618.1** | **300** |
| **2024** | **108** | **40.1** | **287** |
| **2024** | **109** | **0.7** | **5** |
| **2024** | **110** | **800.0** | **390** |
| **2024** | **112** | **598.7** | **363** |
| **2024** | **113** | **139.2** | **67** |
| **2024** | **114** | **791.5** | **357** |
| **2024** | **115** | **320.7** | **230** |
| **2024** | **116** | **312.3** | **177** |
| **2024** | **118** | **759.7** | **410** |
| **2024** | **119** | **758.5** | **411** |
| **2024** | **120** | **169.9** | **77** |
