# Supplementary material for: Spatial overlap of sea ice-associated predators and prey in western Hudson Bay
Source: PLoS One. 2026 Feb 2;21(2):e0328953. doi: 10.1371/journal.pone.0328953 (PMC12863486; doi:10.1371/journal.pone.0328953)
Supplement: S2 Table — These numbers are provided for context and cannot be used as quantitative evidence: given the nature of the observations, seal availability cannot be estimated, and these raw numbers cannot be converted into interpretable index of hunting effort per seal species. (DOCX) [file pone.0328953.s002.docx]

**S2 Table.** Count of seal carcasses from bearded seals, ringed seals, or unknown species with or without distinctive ice features nearby. These numbers are provided for context and cannot be used as quantitative evidence: given the nature of the observations, seal availability cannot be estimated, and these raw numbers cannot be converted into interpretable index of hunting effort per seal species.

| **Seal kill type** | **2019** | **2022** | **2023** | **2024** | **Total** |
| --- | --- | --- | --- | --- | --- |
| **Ringed Seal** | 3 | 7 | 6 | 11 | 27 |
| **Bearded Seal** | 1 | 3 | 2 | 1 | 7 |
| **Unknown at breathing hole** | 0 | 0 | 1 | 0 | 1 |
| **Unknown at crack or lead** | 0 | 15 | 4 | 2 | 21 |
| **Unknown at subnivean lair** | 0 | 3 | 0 | 3 | 6 |
| **Unknown, no distinctive ice feature** | 2 | 13 | 12 | 13 | 40 |
