# Supplementary figures and images for: Spatial overlap of sea ice-associated predators and prey in western Hudson Bay
Source: PLoS One. 2026 Feb 2;21(2):e0328953. doi: 10.1371/journal.pone.0328953 (PMC12863486; doi:10.1371/journal.pone.0328953)

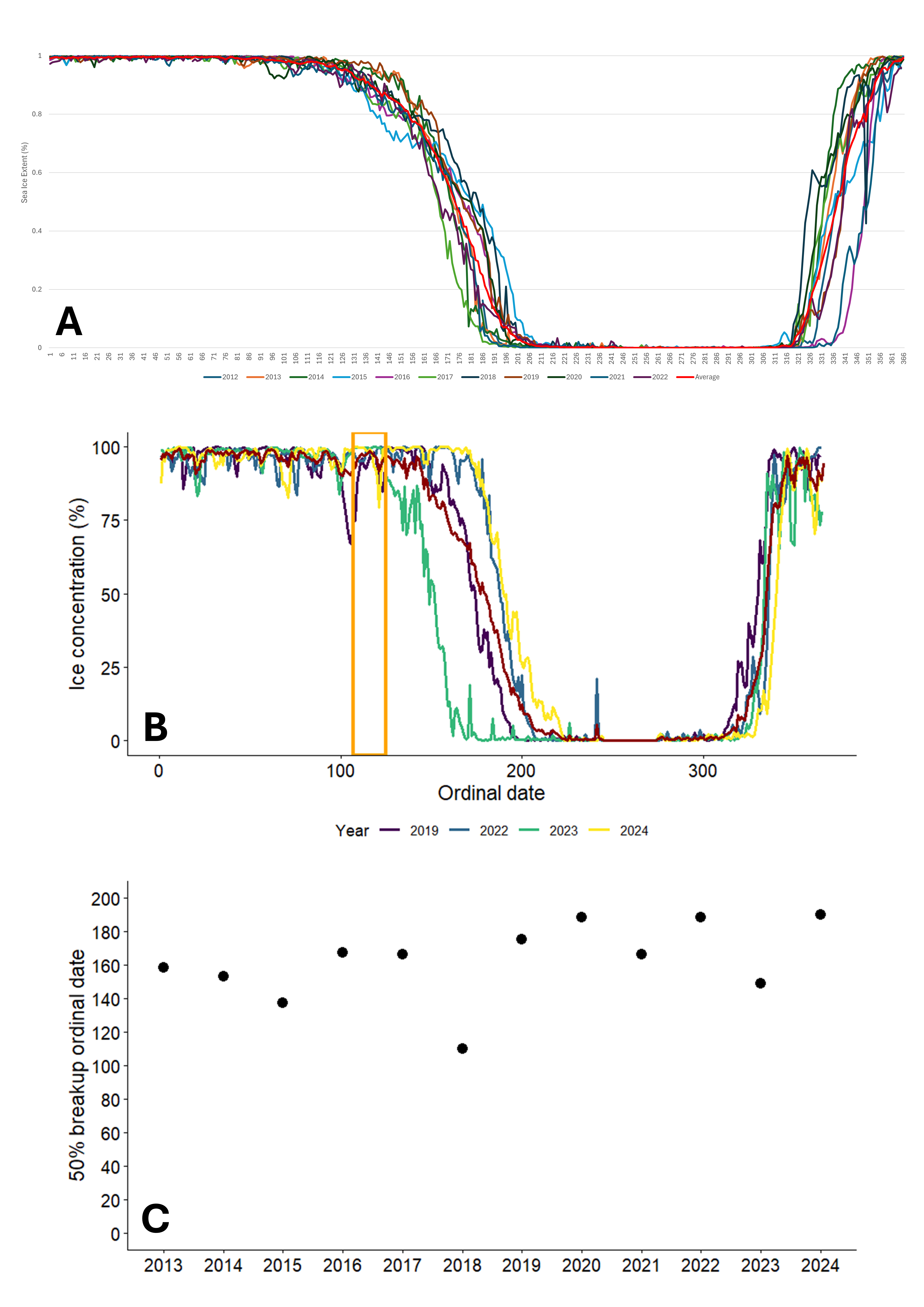

Supplement: S1 Fig — plot (A) shows the daily average for the entire Hudson Bay from 2012 to 2022, with the red line indicating the mean concentration for each ordinal date across all years, plot (B) shows the same metrics for our survey area during the survey period (Table 1, Main Text); the red line again indicates the across-year mean, and the yellow frame highlights the earliest and latest ordinal dates of our surveys. Plot (C) displays the ordinal date of 50% ice breakup for the survey area from 2013 to 2024. (PNG) [file pone.0328953.s003.png]

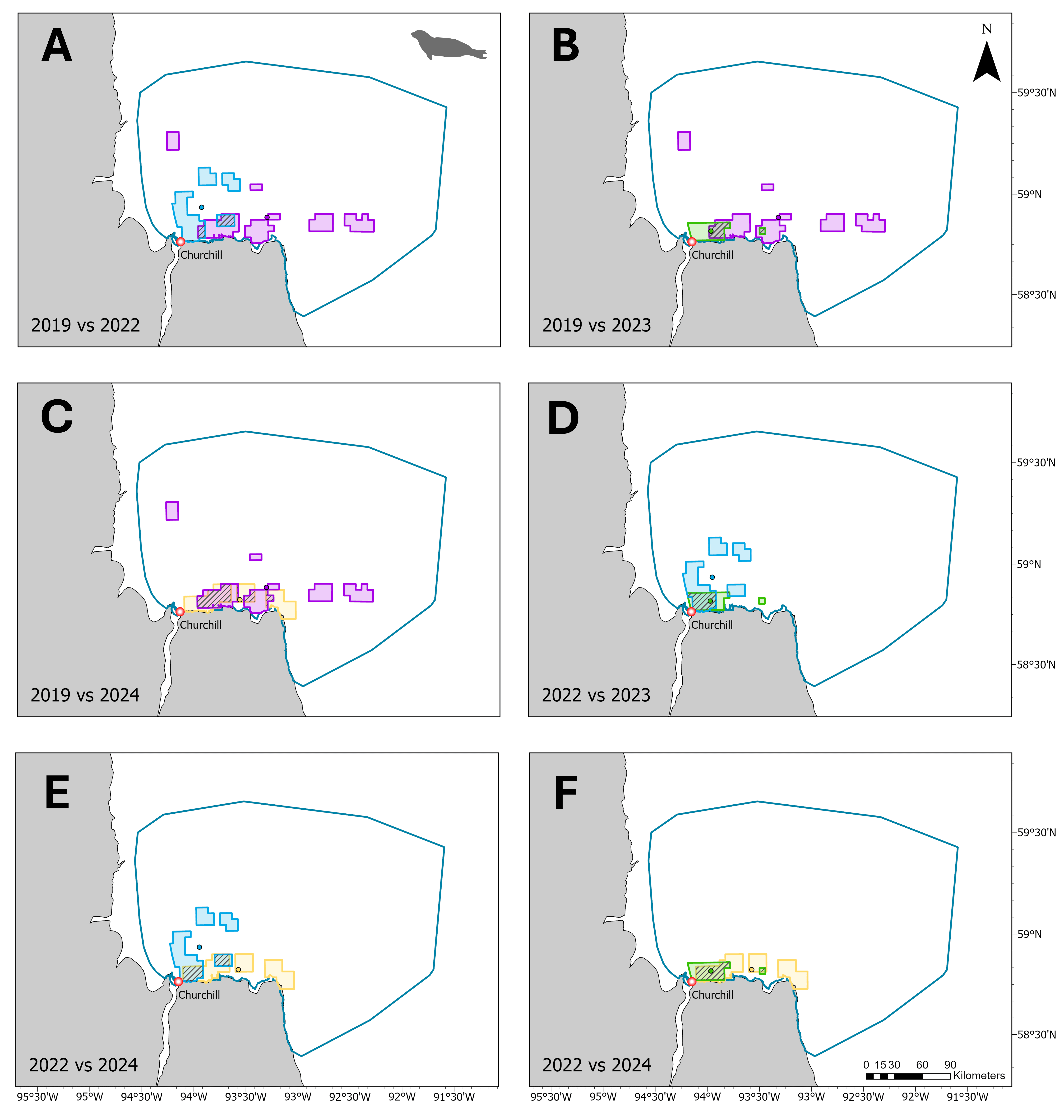

Supplement: S2 Fig — Hotspots were calculated using the Getis-Ord Gi* statistic within the area common to the 2 years compared. We extracted the hotspot area statistically significant at α ≤ 0.05 to produce the overlaps. (PNG) [file pone.0328953.s004.png]

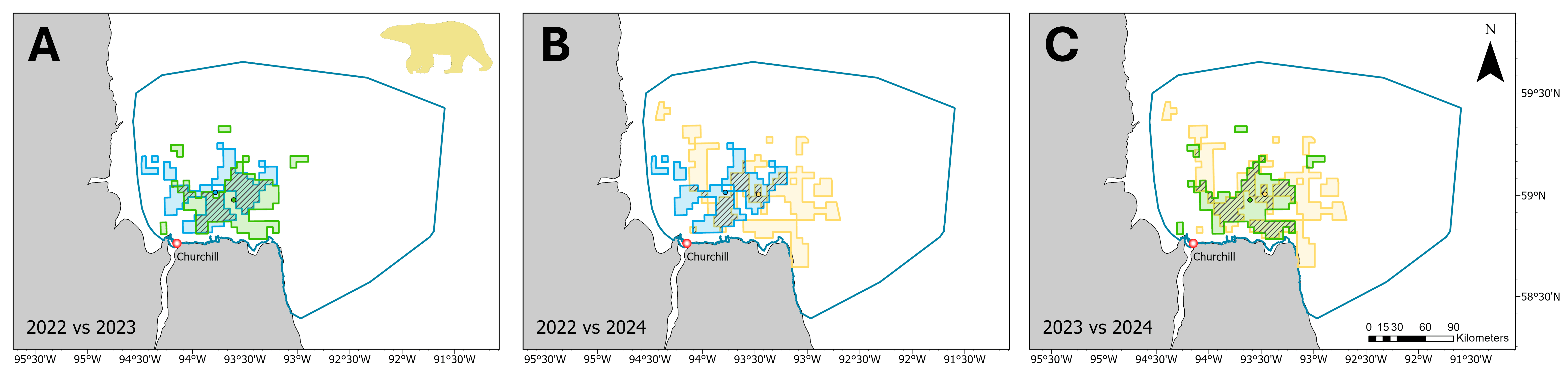

Supplement: S3 Fig — We extracted the hotspot area statistically significant at α ≤ 0.05 to produce the overlaps. (PNG) [file pone.0328953.s005.png]

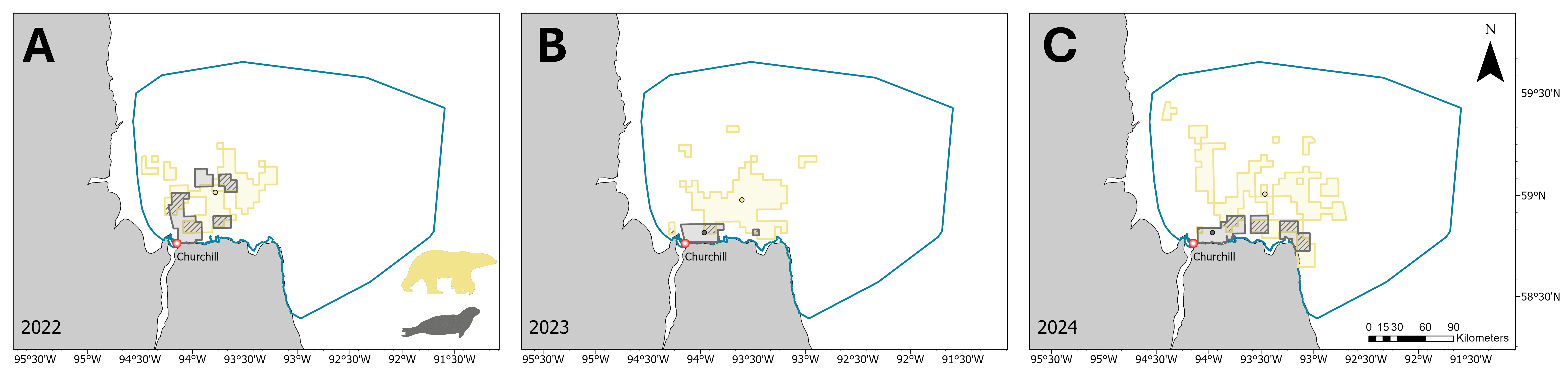

Supplement: S4 Fig — Seals are depicted in grey shades and polar bears in yellow shades, following the convention used in figures from the main text. Hotspots were calculated using the Getis-Ord Gi* statistic. We extracted the hotspot area statistically significant at α ≤ 0.05 to produce the overlaps. (PNG) [file pone.0328953.s006.png]

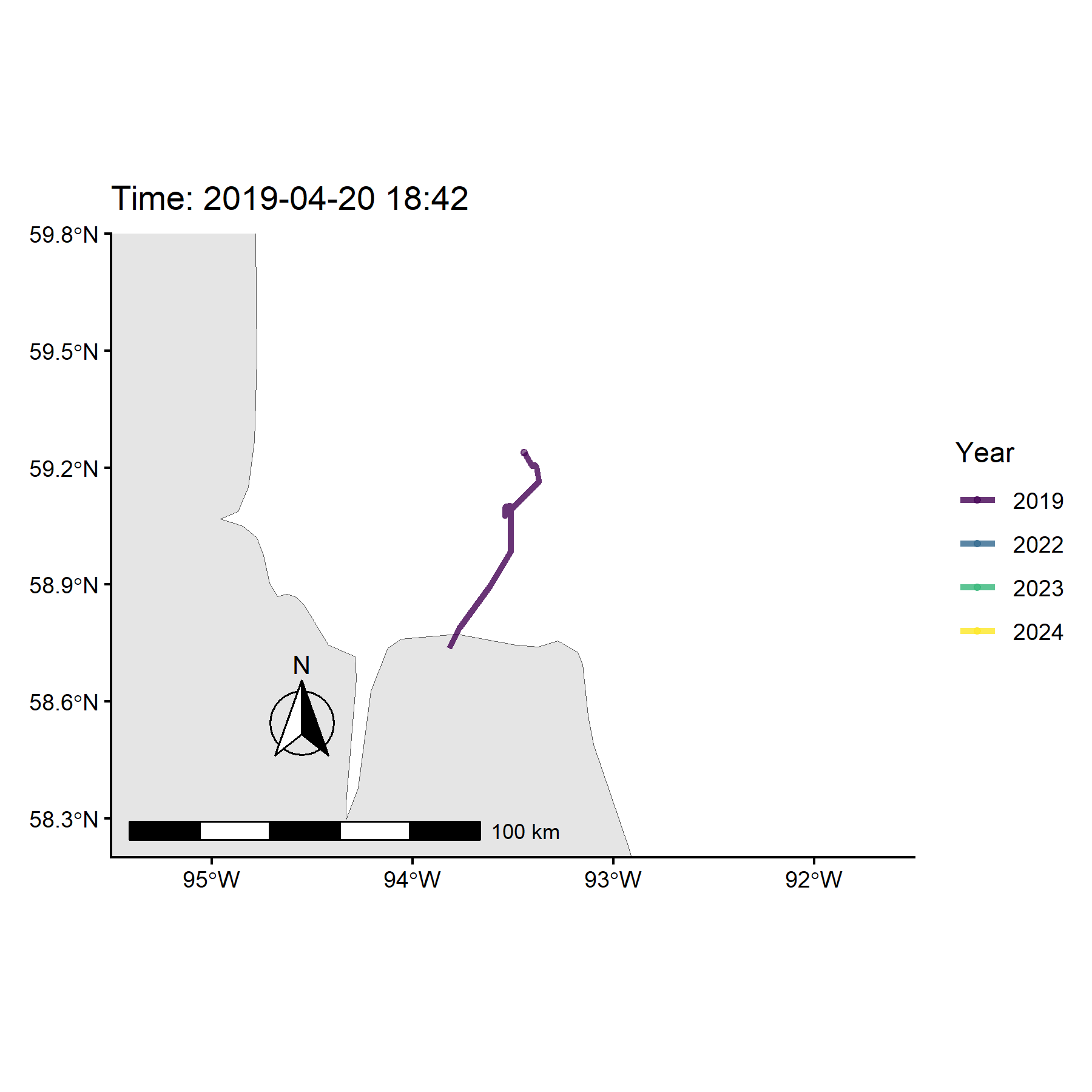

Supplement: S1 Data — Animated gif showing the helicopter survey paths over the study area accumulating over the four years surveyed (blue 2019 green 2022 purple 2023 yellow 2024). (GIF) [file pone.0328953.s007.gif]
