# Supplementary material for: Spatial overlap of sea ice-associated predators and prey in western Hudson Bay
Source: PLoS One. 2026 Feb 2;21(2):e0328953. doi: 10.1371/journal.pone.0328953 (PMC12863486; doi:10.1371/journal.pone.0328953)
Supplement: Attachment — Submitted filename: Review.docx [file pone.0328953.s009.docx]

**Summary:** The authors used visual observations of polar bears, Arctic foxes, seals, kills, and hunting attempts in Western Hudson Bay to produce hotspots of each observation type and to quantify overlap between them. Information on predator-prey interactions in this ecosystem is limited, making this a valuable dataset. Overall I enjoyed reading this paper, but I felt that some of the methods choices needed significant clarification, justification, and/or reconsideration. A more thorough explanation of what the authors did and why would be extremely helpful. Specific areas of concerned are outlined below.

**Hunting attempts versus kills:** Please clarify whether your “hunting attempts” category also included successful kills. Your description in the Methods section (lines 154-160) and your Figure 2 caption make it sound as though your “hunting attempts” and “kills” categories are mutually exclusive (i.e., hunting attempts do not include kills). Maybe I have misunderstood and your hunting attempt category does include successful kills (which is the case in Pilfold et al. 2014), but if that is the case please clarify. Unless you are specifically interested in *UN*successful hunting attempts for some reason that is currently not articulated in the paper, it seems to me that the hunting attempts category *should* include observations of kills. Each kill is inherently a successful hunting attempt, so having a hotspot for *all* hunting attempts (both successful and unsuccessful) seems like it would be a more ecologically meaningful representation of polar bear foraging effort. I also suspect this would change the location of the hunting attempt hotspot, which currently (and a bit nonsensically) has little overlap with the kill hotspot. To be clear, I do see the value in producing a kill hotspot as you have currently done. But I think analyzing your dataset to produce one hotspot for all hunting attempts (both successful and unsuccessful) and one for kills (a subset of those hunting attempts) would be more valuable than how the observations are currently split.

Alternatively, if you felt it was inappropriate to combine kills and unsuccessful hunting attempts due to differences in detectability (e.g., kills being easier to see due to blood on the ice, or not being able to detect unsuccessful attempts at hunting bearded seals), then this should be explained in the text. It would also help to clarify in the Methods at lines 155-158 that hunting attempts were specifically *un*successful hunting attempts -- the reader currently has to read between the lines a bit.

Keeping with the theme of being confused about the intent behind the “hunting attempts” hotspot… You describe observations of hunting attempts as being based on the presence of polar bear tracks at ringed seal lairs and breathing holes, so I guess it is specific to ringed seals. It would be helpful to be explicit about this, either by saying hunting attempts refer specifically to hunting attempts at ringed seal structures, and/or by adding a sentence clarifying that you don’t expect unsuccessful attempts at catching bearded seals to be detectable.

Additionally, in some places (e.g., line 307-309) you seem to use the hunting attempts hotspot as a proxy for the distribution of ringed seal lairs. However, your Methods text makes it clear that you also counted hunting attempts at breathing holes. Please indicate in the Results what percentage of hunting attempt observations were at lairs versus breathing holes. If your motivation for the hunting attempts hotspot was to have a proxy for ringed seal lairs, then perhaps that category should be limited to observations from lairs only.

**Kills:** You note in the Introduction that foxes can kill young ringed seal pups. However, it sounds like all seal kills in your dataset were attributed to polar bears. If this was based on the presence of polar bear tracks at kills, or seal carcasses being too large to have been killed by a fox, it would be good to specify that in the text. Did you have any observations of kills at ringed seal lairs that could have been from foxes? E.g., an excavated lair with blood and no polar bear tracks?

I am also curious about your choice to pool kills by seal species. I understand your point that carcasses may not always be identifiable to species. In addition to the carcass itself, however, it seems like the presence of a breathing hole or lair (as you describe for your “hunting attempts” detection method) could be used to inform species ID at some kill sites. It would be valuable to provide some information in the Results section or in a supplementary table on how many kills could be identified as ringed seals, bearded seals, or unknown, and/or the proportion of kills that occurred at breathing holes and lairs (and thus probably ringed seals) versus at a floe edge (and thus more ambiguous whether the kill was a ringed or bearded seal). This would help support your conclusions about polar bear foraging strategy and predator-prey relationships in the Discussion.

**Abstract**

- Line 25: Rephrase to “Hunting attempts on ringed seal lairs were distributed throughout the study area but ringed seals mostly hauled out on nearshore landfast ice.” Or “Ringed seal lairs were distributed throughout the study area but hauled-out ringed seals were mostly on nearshore landfast ice.” As currently written, the sentence implies that “lairs” were hauling out instead of seals.

**Introduction**

- Line 41: Change “shape” to “shaping” for consistency with “affecting”
- Lines 43-50: If you want to include this information about different types of predator avoidance strategies, and spatial overlap being evidence of whether the prey’s response prevails versus the predator’s response prevails, then you should either identify hypotheses that fit within this framework or circle back to this concept somewhere in your Discussion. It’s currently unclear how these concepts connect to the prey species in your studies, e.g., whether you expect ringed seals and bearded seals to be proactive or reactive, mobile or immobile, etc. You list lairs as being a trait of immobile species that are likely to respond reactively, but at this point in the Introduction you haven’t introduced that ringed seals use lairs so the connection is not clear.
- Line 76 “In addition to scavenging…”: Consider breaking this up into two sentences. It’s currently a bit clunky, with lots of parts and multiple ideas introduced.
- Line 87 “haul out”: suggest replacing with “hauling out onto the sea ice” since some readers may not know what haul out means
- Line 93: Rephrase to “In contrast, bearded seals pup on the surface of the sea ice and do not maintain breathing holes, thus relying on natural sea-ice openings…” Currently, it’s unclear if you are saying bearded seals pup on the surface or if they do not pup on the surface.
- Line 106 “Since most information comes from hauled-out individuals”: Unclear if this is still referring to information from aerial surveys or all information on the distributions of ringed and bearded seals. There’s also some misunderstanding of haul-out here that appears throughout the rest of the paper. Ringed seals inside lairs are still hauled out – they are just hauled out inside a lair instead of on the surface of the of the ice and snow. So aerial surveys are only able to detect *some* hauled out individuals, as individuals hauled out inside of lairs are hidden under the snow and not able to be detected.
- Line 117 “We further hypothesize”: change to past tense for consistency with the rest of the paragraph

**Methods**

- Lines 127-133: It would be helpful to provide some more context for the sea-ice environment within your study area. During the spring, is your entire study area typically covered in sea ice or are there typically large polynyas? What are typical sea-ice concentrations for the area? You give a 90% value for Hudson Bay in general, but it’s not clear if that is representative of your study area, which is a small portion of Hudson Bay.
- Lines 138-150: more information is needed on the survey design and the spatiotemporal distribution of survey effort, whether in written form or via a figure showing helicopter track lines. Were all grid cells plotted in Fig 2 covered by the helicopter or were there gaps in coverage? Were tracks random or systematic? Was there even coverage across the study area in space and time, or were some regions only covered at the start of the survey period, and some only at the end? Were you specifically targeting areas that you expected to have high densities of animals? Please expand.
- Line 139: it’s unclear what “focusing on polar bear captures” means. Do you mean visual captures/observations? Or were these flights conducted to physically capture polar bears as part of another study, with observations made along the way?
- Line 142: delete comma after “species”
- Line 145: rephrase to “Flights occurred when cloud cover was minimal and winds were < 30 km/h.”
- Lines 154-160: Related to my general comments above, please expand and clarify this paragraph. E.g., whether kills counted as hunting attempts, whether hunting attempts and kills included any hunting attempts and kills made by foxes, whether observations of polar bear tracks were treated the same as direct observations of individual bears, etc. This is an important place to be thorough. It’s confusing that you mention individual polar bears here, but then Table 2 only includes polar bear tracks. Were direct observations of polar bears not used in your hotspot analysis, then? Or did the polar bear hotspot include direct observations as well as tracks?
- Line 157: change “seal” to “seals”
- Line 159 “In 2022, we added…”: clarify that this wasn’t ONLY done in 2022, e.g., “In 2022-2024, we also recorded…”
- Line 164 “to match the sea ice data”: You haven’t introduced any sea ice data at this point in the paper
- Line 181: Here or somewhere in this section, before diving into equations, introduce that you chose to do two different types of overlap calculations (a mutual calculation of overlap between species, and directional overlap for the predation-related observations) and why. For Arctic foxes and polar bears, for example, it seems like directional would’ve been appropriate because foxes depend on polar bears but not vice versa. The overlap proportion for bears and foxes is 0.49, but this doesn’t convey that the fox hotspot is almost entirely (just eyeballing Figure 3, >80%) within the bear hotspot, suggesting a strong dependence on polar bears. It’s also not clear to me if the equations you used to calculate overlap are considered standard practice (in which case a citation would be helpful) or if they are something you developed for this paper.
- Line 201 “directional metrics of overlap”: meaning what? How does this differ from what you did for the other species relationships analyses? Please clarify, e.g., “Specifically, we…”
- Line 202 “We quantified the…”: this sentence is hard to follow

**Results**

- Line 219: how did you count tracks, i.e., what counts as one track versus two tracks, etc. in a grid cell? If you see a long line of tracks parallel to your flight path, is that one track?
- Lines 220 “Hotspots were identified for all species and seal kill locations”: why isn’t the hunting attempt hotspot mentioned here, when you did identify a hunting attempts hotspot in your analysis?
- Line 241 “0.95 of the total hotspot area”: use a percentage for consistency with the other paragraphs in this section
- Lines 242-249: Fig 2C includes a broad coldspot area, which would be interesting to mention here (with the caveat that it was only significant at p < 0.1).
- Line 250 “the Arctic fox hotspot covered most of the ice near shore”: this phrasing seems odd, since the ringed seal hotspot has similar coverage of the nearshore ice, and it appears in Fig 2 that there’s nearshore ice in the southern part of the study area southeast of Churchill that the foxes didn’t use.
- Line 252: Present the hotspot area before the landfast ice percentage to better match the order for the other species.

**Discussion**

- Polar bear diet and habitat selection has been shown to vary by sex and age class, at least for some subpopulations. Do you have a sense (either from your surveys or from previous studies in the region) of whether the polar bear observations in this study included all sexes and age classes? Did you observe any cubs? Commenting on this somewhere in the Discussion would add useful context to your conclusions about foraging strategies.
- Line 307-309 “the centroid of the hunting attempt hotspot, representing predatory behavior of bears on ringed seals and therefore reflecting the distribution of ringed seal lairs”: this ignores detection bias, because intact lairs that have not been depredated would not be detectable in your survey flights. Other papers by some of this study’s co-authors acknowledge this, e.g. Pilfold et al. 2014 state that “The observed abundance of pup kills farther from stable ice areas is likely influenced by unequal predatory risk, rather than solely proportional to the abundance of ringed seals breeding in pack ice areas.” Something similar needs to be added to this paper. For this particular sentence, consider replacing “the distribution of ringed seal lairs” with “the distribution of unsuccessful hunting attempts on ringed seal lairs”.
- Line 314 “can breed in the pack ice or give birth in the open…”: A couple points to clarify for this sentence. By “can” do you mean that it occurs, or that it is a viable/successful option? By “breeding” do you mean mating, birthing, nursing?
- Line 352: “still hunting” is a specific polar bear hunting strategy of waiting at seal breathing holes, so suggest rephrasing to “continuing to hunt” or something else
- Line 356 “pupping peak has passed”: please clarify when peak pupping is for your study area and what you mean by “peak pupping”, e.g. whether you mean that the peak of births has passed, or if most ringed seal pups have been weaned by this time, or if most birth lairs are no longer occupied, etc. Currently unclear.
- Lines 368-372 “Arctic foxes also showed high overlap with hauled-out ringed seals…”: I think you’re saying that since peak pupping has passed (again, meaning of “peak pupping” is a bit unclear), any ringed seal pups wouldn’t be newborns and would therefore be too big for Arctic foxes to kill on their own, thus making them dependent on scavenging instead. If that’s the case, it would be good to say it more directly.
- Lines 399-407, detection bias: There are other sources of detection bias present in your study that are important to comment on. For example, intact lairs that have not been depredated would not be detectable in your survey flights, which is important to consider if you are going to attempt to use “hunting attempts” as a proxy for the distribution of ringed seal lairs. I also wondered whether the survey periods included any snowfall events that would obscure kills & predation attempts, potentially unevenly if part of the study area was surveyed right after a heavy snow. It would also be good to acknowledge temporal variability in seal haul-out behavior (which exists for both seal species). This would influence how many seals were observed and, depending on how your survey effort was distributed over time and space, the location of the hotspots identified in your analysis.

**Figures**

- For your hotspot and overlap figures, I kept wondering what portions were landfast ice and what portions were pack ice. I realize the extent and positions of these features can change within and between years, but would it make sense to indicate a representative example in Fig 1, e.g. by adding a line for typical landfast ice extent or landfast ice extent from a given year of your study?.
- Figure 2
  - What does the spatial extent of the grid cells indicate? Is it a minimum convex polygon around all observations of that category? Or what? Please clarify. For Arctic foxes, for example, it looks like the northeast part of the study area wasn’t included in the hotspot analysis and it isn’t clear why. If it’s because there weren’t any fox tracks seen there, then it seems like that should be a cold spot (?).
  - The caption only mentions hotspots, but panel C includes coldspots.
  - Silhouettes: Consider making the silhouettes somewhat to scale (e.g., ringed seal smaller than bearded seal). I also didn’t find the silhouettes for panels E and F very intuitive and suggest considering some alternatives. Examples below for kills (ringed or bearded options, with bear eating the seal) and hunting attempts (same as your current panel F silhouette, but species are slightly more to scale and seal is “escaping”).

- Since these panels have such similar contents, you have an opportunity to reduce visual clutter a bit and make it easier for readers to focus on the actual hotspots. Consider only having the north arrow and scalebar in one of the panels. You could also consider having one legend (potentially off to the side of the figure) instead of having nearly identical legends in all six panels. I also don’t feel that the Hudson Bay label is necessary
- Figure 3 (also largely applicable to Figs S1 and S2)
  - There’s some untapped potential here, and small changes to your plotting choices would go a long way. Because the hotspots are plotted using outlines instead of filled polygons, it’s hard to see where the actual hotspots are. When there’s a hole in a hotspot, for example, the shading within the hotspot and within the hole are currently the same. Areas of overlap are also very hard to see, especially when the hotspots are complex shapes like in FigS2B. These problems could be resolved by using semitransparent filled polygons for each type of hotspot, resulting in a different/darker color where hotspots overlap. These figures are the key result of your paper so I think it’s important that they be strong, intuitive, easy-to-see visualizations of overlap.
  - The suggestions about visual clutter in Figure 2 also apply here (e.g., no need to have a north arrow on every single panel). Reducing clutter will make it easier for readers to key in on the message you are trying to convey with the figure.
- Figures S1 & S2: Year labels like “2019-2022” imply observations from 2019, 2020, 2021, 2022 which is not the case. Perhaps just use a comma instead of a dash.
- Figure S2: I suggest doing something more to differentiate the bears vs seals. Could do column-wise (bear panels all in the left column, seals all in the right column), or split into separate figures.

**Tables**

- Table 1: please convert the “Time flown” column from minutes to hours

I hope these comments are helpful and I wish the authors the best of luck.
