# Supplementary material for: Spatial overlap of sea ice-associated predators and prey in western Hudson Bay
Source: PLoS One. 2026 Feb 2;21(2):e0328953. doi: 10.1371/journal.pone.0328953 (PMC12863486; doi:10.1371/journal.pone.0328953)
Supplement: Attachment — Submitted filename: Response to reviewers.docx [file pone.0328953.s011.docx]

PLOSone: Submission ID PONE-D-25-36545

Dear Dr. Fujiwara,

We are pleased to submit a revised version of our manuscript entitled: " Spatial overlap of sea ice-associated predators and prey in western Hudson Bay ". We thank you and the reviewers for the positive comments on our manuscript and for providing greatly appreciated feedback.

We have attached a marked version of the manuscript ("Revised Manuscript with Track Changes") where changes are highlighted with Word’s Track-changes function. We also attached a document entitled "Response to Reviewers". In this document, we carefully addressed all comments and suggestions proposed by the reviewers. The revised version of our manuscript was read and approved by all authors.

Please note that we updated the list of funders in the acknowledgement section, as some of the original list only contributed to the bear captures *per se*. The funders had no role in study design, data collection and analysis, decision to publish, or preparation of the manuscript. The correct list of funder is “CWR is grateful for postdoctoral financial support from Polar Bears International and MITACS. Research support was provided by the Banrock Station Environmental Trust, Canadian Association of Zoos and Aquariums, Canadian Wildlife Federation, Environment and Climate Change Canada, Churchill Northern Study Center, Hauser Bears, Zuest Family Foundation, Manitoba Agriculture and Resource Development, Natural Sciences and Engineering Research Council of Canada, Polar Bears International, Polar Continental Shelf Program Canada, Quark Expeditions, San Diego Zoo Wildlife Alliance, and World Wildlife Fund Canada.”.

As per PLOS One’s instructions, all figures from the main text were formatted in PACE. Concerning point 5 of additional requirements, the background maps used in the figures are from Naturalearth (<https://www.naturalearthdata.com/>), which as stated in the first lines of their home page “**is free for use in any type of project**” because “**Natural Earth is a public domain map dataset**”. In addition, as stated in the terms of use found on the website (link above): “**No permission is needed to use *Natural Earth.* Crediting the authors is unnecessary.**”. Since there are no concerns about copyright or proprietary data, we have not changed the maps.

Sincerely,

Chloé Warret Rodrigues (on behalf of the co-authors)

University of Manitoba

**MS-ID: PONE-D-25-36545** “Spatial overlap of sea ice-associated predators and prey in western Hudson Bay”

**Academic Editor: Dr. Masami Fujiwara**
**AE-1:** Thank you for submitting your manuscript to PLOS ONE. After careful consideration, we feel that it has merit but does not fully meet PLOS ONE’s publication criteria as it currently stands. Therefore, we invite you to submit a revised version of the manuscript that addresses the points raised during the review process.

**Response:** We are grateful to the Academic editor for his time and support.

**AE-2:** The reviewer finds the dataset valuable and the manuscript generally enjoyable but notes that the Methods require substantial clarification, justification, and in some cases reconsideration. Key concerns include the definitions and treatment of “hunting attempts” and “kills,” the clarity of survey design and data handling, potential detection biases, and figure/table presentation. Numerous minor wording changes are also suggested for clarity and accuracy.

I also find that the **Methods** section lacks sufficient detail. Much greater clarity is needed on survey effort and design, observation categories, and data processing/analysis. A fuller discussion of detection bias is warranted.

**Response:** We thank the Academic editor for his suggestions. Reviewer 2 identified areas where our methods were not clearly communicated. We have consequently clarified (and justified) our definitions of observation categories, updated several category names to avoid ambiguity, and provided additional details on the statistical analyses for readers unfamiliar with hotspot analyses in R (see response to AE-4 and R2-2). These changes should make the Methods fully transparent and reproducible.

We further fleshed out the discussion of detection bias in the Methods and Discussion.

**AE-3:** In addition, because multiple statistical tests are conducted at α < 0.05, adjustments should be made using an approach such as the Bonferroni correction.

**Response:** The hotspot_gistar() function in the *sfhotspot* package already adjusts p-values using inherited method from R::stats through function p.adjust(). We selected the Holm procedure, which strongly controls the family-wise error rate and is thus more conservative than False Discovery Rate-based methods while being more powerful than the Bonferroni adjustment to detect true effects while keeping false positives under control. By using this method, we are confident that if anything, our results are more at risk to have false negative, rather than false positive. We added a sentence reporting the internal use of the Holm procedure (L.233-236): “*Each cell’s Gi* value was associated with a p-value adjusted using the Holm procedure, which effectively controls the family-wise error rate while remaining more powerful than Bonferroni and more conservative than* false discovery rate *methods, thereby balancing the risks of false positives and false negatives.*”

**AE-4:** All p-values should be reported explicitly, rather than simply stating that they are below a significance threshold.

**Response:** We can unfortunately not provide exact p-values, because the hotspot analysis produces 1 p-value per cell. The result of the function hotspot_gistar() from package *sfhotspot* is a tibble with 1 row per cell storing the Gi* value, the associated adjusted p-value and the polygon geometry inherited from package *sf*. The way hotspots are then extracted relies on a user-defined combination of Gi* value and a selected alpha threshold, such as the code snippet below:

AF_gistar <- df_near %>% #input dataset

st_transform("EPSG:32615") %>% #transform CRS

filter(Observation == "Arctic Fox Track") %>% #filter observation of interest

hotspot_gistar(cell_size = 3250, bandwidth = 8000, weights = n) #compute Gi* cell size ~10Km^2^ and bandwidth = 8km.

AF_gistar<-AF_gistar %>%

mutate(class = as.factor(case_when(

gistar>0 & pvalue <0.01 ~ "Gi*>0 p<0.01»,

gistar>0 & pvalue <0.025 ~ " Gi*>0 p<0.025 ",

gistar>0 & pvalue <0.05 ~ " Gi*>0 p<0.05",

TRUE ~ "Insignificant"))) #Extraction of hotspot at different alpha threshold

Consequently, we cannot provide a more precise p-value than our selected threshold. Furthermore, while we fully agree that explicitly reporting p-values is important for statistical models such as regressions, in the context of spatial hotspot analysis, reporting the p-value associated with each grid cell would be of limited interpretive value. The Gi* statistic is intended to identify patterns of clustering at a user-defined geographic scale, rather than testing individual cell-level hypotheses. Presenting per-cell p-values would likely overwhelm the reader with inconsequential numbers while obscuring the broader spatial patterns, which are the primary focus of this analysis.

**AE-5:** The current Data Availability Statement may not meet the journal’s requirements; please review the journal’s data availability policy carefully and revise accordingly.

**Response:** The data will be stored in Mendeley Data with reserved DOI: 10.17632/3pggzffmj8.1. We apologize for using a placeholder statement during submission while we finalized our choice of repository.

**Reviewer 1: Dr. Lori Quakenbush**

**R1-1:** This manuscript is well written and describes spatial overlap of four ice associated species, which is difficult to achieve. I have edited a Word document in track changes but found few things to comment on. I recommend an additional reference to be considered. Once these minor issues are considered this manuscript should be accepted for publication.

**Response:** We are grateful to the reviewer for the positive comments and suggestions.

We summarize our changes below. Line numbers refer to the original manuscript with corresponding lines in the “Revised Manuscript with Track Changes” between parentheses.

**L.24-25:** Our sentence read as if the lairs were hauling out, as Reviewer 2 also noticed. We rephrased as follows: “Ringed seals built lairs throughout the study area but they mostly hauled out on landfast ice.” (L.25-26)

**L.62-63:** All citations converted to numbers and numbers re-ordered to match the order of citation. Thank you.

**L.74:** “because” added

**L.127:** “after which” added

**L.152:** “s” added

**L.217:** reference to coldspots removed (see response to R2-27 and R2-38). However, we added L.198 that hotspots are clusters of high values, and coldspots clusters of low values.

**L.218:** We use brackets to indicate ranges because, following mathematical convention, they denote closed intervals (i.e., endpoints are included in the range). We also fixed the space inconsistency when reporting mean ± SE throughout the text (thank you for noticing).

**L.244:** Indeed, this figure reference should be S2! Corrected. Thank you for catching this typo.

**R1-2:** L.335-338: But see Rode et al. 2020 DOI: 10.1111/gcb.15572 Adult male bears found to consume more bearded seals than other age and sexes.

**Response:** Intrapopulation variation in diet has been documented in multiple polar bear populations. In the Chukchi Sea, Rode et al. (2021) indeed reported that adult males consumed higher proportions of bearded seals than other demographic groups, a pattern also observed in earlier studies from other regions (e.g., Thiemann et al. 2007, 2008). In contrast, Galicia et al. (2015) found that adult females in Baffin Bay consumed more bearded seals than adult males. Despite this intrapopulation variability, ringed seals remained the primary prey at the population level in all studies, including Rode et al. (2021). Our statement focused on this population-level trend rather than intrapopulation variation.

We added Rode et al. (2021) to our citations, because it is relevant literature confirming the importance of bearded seals to polar bear diet, and was missing (L.386).

References:

Thiemann GW, Budge SM, Iverson SJ, Stirling I (2007). Unusual fatty acid biomarkers reveal age- and sex-specific foraging in polar bears (Ursus maritimus). Can J Zool 85: 505–517

Thiemann GW, Iverson SJ, Stirling I (2008). Polar bear diets and Arctic marine food webs: insights from fatty acid analysis. Ecol Monogr 78: 591–613

Galicia MP, Thiemann GW, Dyck MG, & Ferguson SH (2015). Characterization of polar bear (Ursus maritimus) diets in the Canadian High Arctic. *Polar Bio* *38*: 1983-1992

**L.343:** Noted: “still” replaced with “also” to avoid confusion.

**R1-3:** L.371: This is a ringed seal paper.

**Response:** We apologize for the confusion. In an earlier version, the statement compared bearded seals to ringed seal pups. We reverted to comparing the two species and separated the citations: “The large size of bearded seal pups at weaning [1] in comparison to ringed seal pups [2,3] likely prevents direct predation by foxes.” (L431-432).

1. Kovacs KM, Krafft BA, Lydersen C. Bearded seal (*Erignathus barbatus*) birth mass and pup growth in periods with contrasting ice conditions in Svalbard, Norway. Mar Mammal Sci. 2020;36: 276–284. doi:10.1111/mms.12647

2. Hammill MO, Lydersen C, Ryg M, Smith TG. Lactation in the ringed seal (*Phoca hispida*). Can J Fish Aquat Sci. 1991;48: 2471–2476. doi:10.1139/f91-288

3. Smith TG, Hammill MO, Taugbol G. A review of the developmental, behavioural and physiological adaptations of the ringed seal, Phoca hispida, to life in the Arctic winter. Arctic. 1991;44: 124–131. doi:10.14430/arctic1528

**Reviewer 2:**

**General comments**

**R2-1:** The authors used visual observations of polar bears, Arctic foxes, seals, kills, and hunting attempts in Western Hudson Bay to produce hotspots of each observation type and to quantify overlap between them. Information on predator-prey interactions in this ecosystem is limited, making this a valuable dataset. Overall, I enjoyed reading this paper, but I felt that some of the methods choices needed significant clarification, justification, and/or reconsideration. A more thorough explanation of what the authors did and why would be extremely helpful. Specific areas of concern are outlined in the attached document along with line-specific comments.

I had to select "no" for the data availability question in the reviewer form because the PLOS One Data Availability section in the manuscript pdf specifically says that "stating 'data available on requestion from the author' is not sufficient" except in an "exceptional situation".

**Response:** We thank the reviewer for the support and for the useful suggestions. We review each comment below.

See response to AE-4: The data will be archived in Mendeley Data with Reserved DOI: 10.17632/3pggzffmj8.1. We changed our statement accordingly.

**R2-2:** Hunting attempts versus kills: Please clarify whether your “hunting attempts” category also included successful kills. Your description in the Methods section (lines 154-160) and your Figure 2 caption make it sound as though your “hunting attempts” and “kills” categories are mutually exclusive (i.e., hunting attempts do not include kills). Maybe I have misunderstood and your hunting attempt category does include successful kills (which is the case in Pilfold et al. 2014), but if that is the case please clarify. Unless you are specifically interested in *UN*successful hunting attempts for some reason that is currently not articulated in the paper, it seems to me that the hunting attempts category *should* include observations of kills. Each kill is inherently a successful hunting attempt, so having a hotspot for *all* hunting attempts (both successful and unsuccessful) seems like it would be a more ecologically meaningful representation of polar bear foraging effort. I also suspect this would change the location of the hunting attempt hotspot, which currently (and a bit nonsensically) has little overlap with the kill hotspot. To be clear, I do see the value in producing a kill hotspot as you have currently done. But I think analyzing your dataset to produce one hotspot for all hunting attempts (both successful and unsuccessful) and one for kills (a subset of those hunting attempts) would be more valuable than how the observations are currently split.

Keeping with the theme of being confused about the intent behind the “hunting attempts” hotspot… You describe observations of hunting attempts as being based on the presence of polar bear tracks at ringed seal lairs and breathing holes, so I guess it is specific to ringed seals. It would be helpful to be explicit about this, either by saying hunting attempts refer specifically to hunting attempts at ringed seal structures, and/or by adding a sentence clarifying that you don’t expect unsuccessful attempts at catching bearded seals to be detectable.

Additionally, in some places (e.g., line 307-309) you seem to use the hunting attempts hotspot as a proxy for the distribution of ringed seal lairs. However, your Methods text makes it clear that you also counted hunting attempts at breathing holes. Please indicate in the Results what percentage of hunting attempt observations were at lairs versus breathing holes. If your motivation for the hunting attempts hotspot was to have a proxy for ringed seal lairs, then perhaps that category should be limited to observations from lairs only.

**Response:** We thank the reviewer for the opportunity to reduce confusion in our readers.

The “hunting attempt” and “seal kill” events are mutually exclusive as recorded in the field. A “hunting attempt” is recorded when a polar bear digs into a ringed seal subnivean lair or snow-covered breathing hole and success is not evident (e.g., no carcass, prey remains or blood). If a hunting attempt on ringed seal resulted in an obvious kill, as indicated by the presence of a carcass and blood, then this event is recorded as “seal kill”.

In our analyses, however, the “hunting attempt” layer was a combination of the “hunting attempt” events as recorded in the field and events from “seal kill” directed at structures built by ringed seals (i.e., the hotspot included both unsuccessful and successful attempts to hunt ringed seals). This hotspot was intended as a conservative proxy for ringed seal activity during winter and spring.

To clarify our intentions and Methods section:

1) We renamed the hotspot “hunting attempt” as “ringed seal structure” (or “structure” for short).
2) We clarified the definitions of all observations, particularly hunting attempt (now structure) and seal kill (L.178-191), and added a paragraph explaining why we use each layer (L.192-205).
3) We added a table with each observation term, its definition, whether it is direct or indirect, and the sample size used to calculate each hotspot (Table 2), as an easy-to-consult tool for readers.
4) Table 3 (formerly Table 2) which indicates the raw count of observations may have created further confusion. While most sets of observations directly correspond to a hotspot, the structure hotspots combined all “hunting attempts” and some “seal kills”. We have added the following statement to Table 3 caption to clarify: “*Raw observation counts per category (fox tracks and bear tracks were not recorded in 2019). 8 seal kills from 2022, 4 from 2023 and 8 from 2024 occurred at lairs or breathing holes and were combined with polar bear digs of lairs and breathing holes to calculate the hotspot “structure”.*”. We also changed “hunting attempt” into self-explanatory “Polar bear dig of lairs and breathing holes”.
5) Hunting attempts are all digging events, but we do not stop at each digging observation; therefore, we cannot always identify the feature that was dug out. Although birth lairs are most likely, male ringed seals can also build resting lairs, and some of these excavations can also be directed at snow-covered breathing holes. We added “snow-covered breathing holes” (L.180) to prevent any confusion.

**R2-2:** Kills: You note in the Introduction that foxes can kill young ringed seal pups. However, it sounds like all seal kills in your dataset were attributed to polar bears. If this was based on the presence of polar bear tracks at kills, or seal carcasses being too large to have been killed by a fox, it would be good to specify that in the text. Did you have any observations of kills at ringed seal lairs that could have been from foxes? E.g., an excavated lair with blood and no polar bear tracks?

**Response:** Thank you for drawing our attention to that matter. Indeed, all kills and digs at lairs were from polar bears (as was indicated L.152 *original manuscript*), which may partly be because 1) at this time of the year there are fewer ringed seal newborn pups, so ringed seal pups are more likely killed by bears than foxes, and 2) the primary goal of these surveys is to capture polar bears to fit them with satellite tags. We thus fly until finding a polar bear track, which we follow to find and capture the bear, recording observations systematically during the whole flight.

We now explicitly wrote that we did not see lairs excavated by foxes (L.182).

**R2-3:** I am also curious about your choice to pool kills by seal species. I understand your point that carcasses may not always be identifiable to species. In addition to the carcass itself, however, it seems like the presence of a breathing hole or lair (as you describe for your “hunting attempts” detection method) could be used to inform species ID at some kill sites. It would be valuable to provide some information in the Results section or in a supplementary table on how many kills could be identified as ringed seals, bearded seals, or unknown, and/or the proportion of kills that occurred at breathing holes and lairs (and thus probably ringed seals) versus at a floe edge (and thus more ambiguous whether the kill was a ringed or bearded seal). This would help support your conclusions about polar bear foraging strategy and predator-prey relationships in the Discussion.

**Response:** We only have 7 observations of kill sites of unidentified seal species with distinctive features that suggested ringed seals. Even if we added these 7 carcass observations to the total count of ringed-seal carcasses, we would still not have enough observations to produce a hotspot (< 40).

We added descriptive information in supplemental table S2 as requested, for transparency. However, while we can standardize the data for spatial analyses, our dataset does not allow reliable estimation of seal availability, so the raw counts of ringed versus bearded seals cannot be converted to meaningful proportions. We also lack comparable data from other periods, which could have allowed quantifying a switch. Additionally, we cannot estimate failed predation events on bearded seals (noted in the table caption). Therefore, we can only offer tentative explanations for the observed spatial patterns, as we had done in the Discussion.

**Abstract**

**R2-4:** Line 25: Rephrase to “Hunting attempts on ringed seal lairs were distributed throughout the study area but ringed seals mostly hauled out on nearshore landfast ice.” Or “Ringed seal lairs were distributed throughout the study area but hauled-out ringed seals were mostly on nearshore landfast ice.” As currently written, the sentence implies that “lairs” were hauling out instead of seals.

**Response:** Rephrased, see response to Reviewer 1 L.25-26.

**Introduction**

**R2-5:** Line 41: Change “shape” to “shaping” for consistency with “affecting”

**Response:** Done (L.42)

**R2-6:** Lines 43-50: If you want to include this information about different types of predator avoidance strategies, and spatial overlap being evidence of whether the prey’s response prevails versus the predator’s response prevails, then you should either identify hypotheses that fit within this framework or circle back to this concept somewhere in your Discussion. It’s currently unclear how these concepts connect to the prey species in your studies, e.g., whether you expect ringed seals and bearded seals to be proactive or reactive, mobile or immobile, etc. You list lairs as being a trait of immobile species that are likely to respond reactively, but at this point in the Introduction you haven’t introduced that ringed seals use lairs so the connection is not clear.

**Response:** We thank the reviewer for the suggestion. We fleshed out the introduction and re-organized it to develop the idea (L.44-62) and circled back to this idea in the Discussion (L.426-440).

**R2-7:** Line 76 “In addition to scavenging…”: Consider breaking this up into two sentences. It’s currently a bit clunky, with lots of parts and multiple ideas introduced.

**Response:** Run-on sentence split: “In addition to scavenging on marine mammal carcasses, Arctic fox prey on newborn ringed seals [37, 38]. They face little interspecific competition in using marine resources because their main competitor, the red fox (*Vulpes vulpes*), does not commonly venture onto the sea ice [39].” (L.88-91)

**R2-8:** Line 87 “haul out”: suggest replacing with “hauling out onto the sea ice” since some readers may not know what haul out means

**Response:** Replaced. (L.99)

**R2-9:** Line 93: Rephrase to “In contrast, bearded seals pup on the surface of the sea ice and do not maintain breathing holes, thus relying on natural sea-ice openings…” Currently, it’s unclear if you are saying bearded seals pup on the surface or if they do not pup on the surface.

**Response:** statements reversed. (L.105-108)

**R2-10:** Line 106 “Since most information comes from hauled-out individuals”: Unclear if this is still referring to information from aerial surveys or all information on the distributions of ringed and bearded seals. There’s also some misunderstanding of haul-out here that appears throughout the rest of the paper. Ringed seals inside lairs are still hauled out – they are just hauled out inside a lair instead of on the surface of the of the ice and snow. So aerial surveys are only able to detect *some* hauled out individuals, as individuals hauled out inside of lairs are hidden under the snow and not able to be detected.

**Response:** True! We rephrased as follow for accuracy: “Because most information comes from individuals hauled-out on top of the ice, little is known about the distribution of ringed seal birth lairs or resting lairs [47,59], with no information available from Hudson Bay.” (L.119-121).

**R2-11:** Line 117 “We further hypothesize”: change to past tense for consistency with the rest of the paragraph

**Response:** Done. (L.130)

**Methods**

**R2-12:** Lines 127-133: It would be helpful to provide some more context for the sea-ice environment within your study area. During the spring, is your entire study area typically covered in sea ice or are there typically large polynyas? What are typical sea-ice concentrations for the area? You give a 90% value for Hudson Bay in general, but it’s not clear if that is representative of your study area, which is a small portion of Hudson Bay.

**Response:** We moved our statement about the major flaw lead to the description of the study area (L.143-145). We are not aware of such index published specifically for the area near Churchill for spring time, but we provided a few more details from reference 51, which is now reference 52 (L.147-149). We also calculated the mean ice concentration within the boundaries of our polygon for the period of our survey using AMSR2-v54 at 3km resolution and provide a supplemental figure (S1 Fig.) with A) Ice concentration vs ordinal date for the whole bay and the whole year for context, B) Ice concentration vs ordinal date for our study area and C) the ordinal date of 50% ice breakup for our study area from 2017 to 2024: “*In comparison to other parts of the Bay, breakup occurs later in western Hudson Bay (including the area near Churchill), and the ice concentration remains high (≥ 5/10) until late in July [52]. Within the boundaries of our study area, the mean ice concentration for the period of our survey (Table 1) was 97.5%±0.4% [50.3% - 100%] (S1 Fig.; calculated using AMSR2-v54 at 3km resolution), and 50% ice breakup typically occurs between the last week of June and the first half of July (S1 Fig).*”

52. Gagnon AS, Gough WA. Trends in the dates of ice freeze-up and breakup over Hudson Bay, Canada. Arctic. 2005;58: 370–382. doi:10.14430/arctic451.

**S1 Fig.** Mean daily sea ice concentration (%) calculated from AMSR2-v54 at 3 km resolution: plot (A) shows the daily average for the entire Hudson Bay from 2012 to 2022, with the red line indicating the mean concentration for each ordinal date across all years, plot (B) shows the same metrics for our survey area during the survey period (Table 1, Main Text); the red line again indicates the across-year mean, and the yellow frame highlights the earliest and latest ordinal dates of our surveys. Plot (C) displays the ordinal date of 50% ice breakup for the survey area from 2013 to 2024.

**R2-13:** Lines 138-150: more information is needed on the survey design and the spatiotemporal distribution of survey effort, whether in written form or via a figure showing helicopter track lines. Were all grid cells plotted in Fig 2 covered by the helicopter or were there gaps in coverage? Were tracks random or systematic? Was there even coverage across the study area in space and time, or were some regions only covered at the start of the survey period, and some only at the end? Were you specifically targeting areas that you expected to have high densities of animals? Please expand.

**Response:** Details added (L.159-161). As stated L.163 of the original draft (now L.218) spatial coverage was uneven, hence we had to standardize the observation data, and pool observations over the 4 years. We added an animated gif of the helicopter track (S1 animation) to display spatio-temporal coverage.

**R2-14:** Line 139: it’s unclear what “focusing on polar bear captures” means. Do you mean visual captures/observations? Or were these flights conducted to physically capture polar bears as part of another study, with observations made along the way?

**Response:** We meant to physically capture bears. Details added (L.159-160). “*The primary goal of the flight survey was to capture individual bears to fit them with satellite tags.*” (L.155-156).

**R2-15:** Line 142: delete comma after “species”

**Response:** “Species” deleted, but comma deleted after “Arctic fox” (L.165).

**R2-16:** Line 145: rephrase to “Flights occurred when cloud cover was minimal and winds were < 30 km/h.”

**Response:** We rephrased that part. (L.169-170)

**R2-17:** Lines 154-160: Related to my general comments above, please expand and clarify this paragraph. E.g., whether kills counted as hunting attempts, whether hunting attempts and kills included any hunting attempts and kills made by foxes, whether observations of polar bear tracks were treated the same as direct observations of individual bears, etc. This is an important place to be thorough. It’s confusing that you mention individual polar bears here, but then Table 2 only includes polar bear tracks. Were direct observations of polar bears not used in your hotspot analysis, then? Or did the polar bear hotspot include direct observations as well as tracks?

**Response:** See response to **R2-1**, **R2-2** and **R2-3**.

We removed the mention of individual polar bears. There were too few to calculate a reliable hotspot, so we did not use them.

**R2-18:** Line 157: change “seal” to “seals”

**Response:** Sentence reworded. (L.183-184)

**R2-19:** Line 159 “In 2022, we added…”: clarify that this wasn’t ONLY done in 2022, e.g., “In 2022-2024, we also recorded…”

**Response:** Fair point! Statement rephrased (L.189).

**R2-20:** Line 164 “to match the sea ice data”: You haven’t introduced any sea ice data at this point in the paper

**Response:** Indeed, we deleted the statement. (L.215-216)

**R2-21:** Line 181: Here or somewhere in this section, before diving into equations, introduce that you chose to do two different types of overlap calculations (a mutual calculation of overlap between species, and directional overlap for the predation-related observations) and why. For Arctic foxes and polar bears, for example, it seems like directional would’ve been appropriate because foxes depend on polar bears but not vice versa. The overlap proportion for bears and foxes is 0.49, but this doesn’t convey that the fox hotspot is almost entirely (just eyeballing Figure 3, >80%) within the bear hotspot, suggesting a strong dependence on polar bears. It’s also not clear to me if the equations you used to calculate overlap are considered standard practice (in which case a citation would be helpful) or if they are something you developed for this paper.

**Response:** Great idea. We thank the reviewer for the suggestion. We added directional fox-bear overlap and moved up the paragraph about directional overlaps (L.245-251). We also added a description of the calculation “*We calculated directional overlaps as the area of overlap between two hotspots divided by the area of the hotspot of interest.*” (L.246-248).

Concerning the equation we proposed: although it is not a named metric, it provides a symmetric and size-weighted measure of overlap between two discrete hotspot polygons. It is conceptually analogous to established overlap indices widely used in ecology, such as the Bhattacharyya coefficient, which provides a symmetric measure of overlap between continuous utilization distributions, or to other indices developed for home-range overlap, such as those developed for continuous UD in Fieberg & Kochanny (2005).

Fieberg, J., & Kochanny, C. O. (2005). Quantifying home‐range overlap: the importance of the utilization distribution. *The Journal of Wildlife Management*, *69*(4), 1346-1359.

**R2-22:** Line 201 “directional metrics of overlap”: meaning what? How does this differ from what you did for the other species relationships analyses? Please clarify, e.g., “Specifically, we…”

**Response:** See Response to R2-21 and R2-23.

**R2-23:** Line 202 “We quantified the…”: this sentence is hard to follow

**Response:** this sentence explains the directional overlap, and was modified in response to R2-21 and AE-2 “*Mutual overlap was calculated for all species pairs, while directional overlap served to quantify the proportion of Arctic fox hotspot overlapped by bear hotspot, of polar bear and seal hotspots overlapped by seal-kill or lair dig hotspots, and the proportion of seal-kill, and lair dig hotspots overlapped by Arctic fox hotspots.*” (L.248-251).

**Results**

**R2-24:** Line 219: how did you count tracks, i.e., what counts as one track versus two tracks, etc. in a grid cell? If you see a long line of tracks parallel to your flight path, is that one track?

**Response:** sentence added “*In 2022-2024, we recorded polar bear and Arctic fox tracks as points, giving each point the coordinates of the initial sighting and treating it as part of the same track until we could no longer follow it.*” (L.189-191)

**R2-25:** Lines 220 “Hotspots were identified for all species and seal kill locations”: why isn’t the hunting attempt hotspot mentioned here, when you did identify a hunting attempts hotspot in your analysis?

**Response:** It was a shortcut because hunting attempt (now termed “structure”) represent ringed seals exclusively. “structure” added (L.285).

**R2-26:** Line 241 “0.95 of the total hotspot area”: use a percentage for consistency with the other paragraphs in this section

**Response:** Thank you for catching the typo! Changed to percentage (L.307)

**R2-27:** Lines 242-249: Fig 2C includes a broad coldspot area, which would be interesting to mention here (with the caveat that it was only significant at p < 0.1).

**Response:** For the reasons explained in Response to R2-38, cold spots should be interpreted with caution. We initially reported the few detected cold spots for transparency: the model identified a few low-significance cold spots near the survey boundaries (0.05 < p < 0.1). Given these high p-values and the non-systematic nature of our surveys, uncertainty around cold spots is too great for reliable interpretation. Following your comment R2-38, we thought it best to remove any mention of cold spots to avoid confusion because we could not reliably discuss these cold spots. Instead, we mentioned that our data does not allow much confidence in absence data, contrary to presence data

“*As flight paths were non-systematic, our data only support inference on sign presence, not absence.* *Therefore, we could identify hotspots but not confidently infer coldspots. Each cell’s Gi* value was associated with a p-value adjusted using the Holm procedure, which controls the family-wise error rate while remaining more powerful than Bonferroni and more conservative than* *false discovery rate methods, thereby balancing the risks of false positives and false negatives.*” (L.231-236)

**R2-28:** Line 250 “the Arctic fox hotspot covered most of the ice near shore”: this phrasing seems odd, since the ringed seal hotspot has similar coverage of the nearshore ice, and it appears in Fig 2 that there’s nearshore ice in the southern part of the study area southeast of Churchill that the foxes didn’t use.

**Response:** We replaced the original statement with “the Arctic fox hotspot covered most of the ice near the northern shore but extended onto the pack ice to the northwest” (L.315-316).

**R2-29:** Line 252: Present the hotspot area before the landfast ice percentage to better match the order for the other species.

**Response:** Sentences reversed. (L.316-318)

**Discussion**

**R2-30:** Polar bear diet and habitat selection has been shown to vary by sex and age class, at least for some subpopulations. Do you have a sense (either from your surveys or from previous studies in the region) of whether the polar bear observations in this study included all sexes and age classes? Did you observe any cubs? Commenting on this somewhere in the Discussion would add useful context to your conclusions about foraging strategies.

**Response:** In this particular data set, we have very few observations of tracks from family groups, but all ages are represented. We’ve added this observation (L.451-453).

**R2-31:** Line 307-309 “the centroid of the hunting attempt hotspot, representing predatory behavior of bears on ringed seals and therefore reflecting the distribution of ringed seal lairs”: this ignores detection bias, because intact lairs that have not been depredated would not be detectable in your survey flights. Other papers by some of this study’s co-authors acknowledge this, e.g. Pilfold et al. 2014 state that “The observed abundance of pup kills farther from stable ice areas is likely influenced by unequal predatory risk, rather than solely proportional to the abundance of ringed seals breeding in pack ice areas.” Something similar needs to be added to this paper. For this particular sentence, consider replacing “the distribution of ringed seal lairs” with “the distribution of unsuccessful hunting attempts on ringed seal lairs”.

**Response:** Fair point. We have added: “*However, lair detection was limited to those excavated by polar bears when they are selecting areas where prey were pupping [74]. We may, thus, have underestimated the use of landfast ice for seal lair construction as this was not a high use area for bears.*” (L.377-380).

**R2-32:** Line 314 “can breed in the pack ice or give birth in the open…”: A couple points to clarify for this sentence. By “can” do you mean that it occurs, or that it is a viable/successful option? By “breeding” do you mean mating, birthing, nursing?

**Response:** Yes, “can” means that it was observed elsewhere (i.e., in Svalbard), as reflected in the references at the end of the sentence. We replaced “breed” by “build birth lairs” (L.384).

**R2-33:** Line 352: “still hunting” is a specific polar bear hunting strategy of waiting at seal breathing holes, so suggest rephrasing to “continuing to hunt” or something else

**Response:** Done (See response to Reviewer 1 L.445).

**R2-34:** Line 356 “pupping peak has passed”: please clarify when peak pupping is for your study area and what you mean by “peak pupping”, e.g. whether you mean that the peak of births has passed, or if most ringed seal pups have been weaned by this time, or if most birth lairs are no longer occupied, etc. Currently unclear.

**Response:** In general pupping is used for “giving birth”, but we have rephrased. L.426 “In Hudson Bay, ringed seal births end in late April” and L.435-436 “*In contrast, bearded seals depend on broken ice, and give birth on the surface of ice near water in late April-May*”.

**R2-35:** Lines 368-372 “Arctic foxes also showed high overlap with hauled-out ringed seals…”: I think you’re saying that since peak pupping has passed (again, meaning of “peak pupping” is a bit unclear), any ringed seal pups wouldn’t be newborns and would therefore be too big for Arctic foxes to kill on their own, thus making them dependent on scavenging instead. If that’s the case, it would be good to say it more directly.

**Response:** No, we just meant the Arctic fox hotspot overlapped with the hotspot derived from hauled-out ringed seals. While one could interpret this overlap as foxes tracking seal presence to prey on pups or scavenge polar bear kills, our surveys occurred near the end of the ringed seal pupping season, and the sharp olfactory sense of foxes combined with lower nearshore bear activity, weakens this interpretation. A possible explanation is that in the absence of detected carcasses, foxes limit their exploratory movements to sea ice closer to shore on easier terrain, or that some days they were constrained to the landfast ice until the flaw lead closed and they could reach the pack ice. We removed the reference to possible pup predation, as it may have caused confusion and was not essential. We also placed the final clause in parentheses, as it was not central to the main point of the sentence, which now reads: “*Arctic foxes also showed high overlap with hauled-out ringed seals (50%).* *Because our surveys occurred after the peak of ringed seal births, and because bear activity is lower on the landfast ice than on the pack ice, this association may reflect a reluctance of foxes to extend exploratory movements too far from shore or onto the riskier pack ice (if no carcass is detected). The flaw lead may also act as a barrier at least part of the time, concentrating fox activity along the landfast ice as they search for passage to the pack ice (i.e., where most bear hunting activity occurs).*” (L.467-473).

**R2-36:** Lines 399-407, detection bias: There are other sources of detection bias present in your study that are important to comment on. For example, intact lairs that have not been depredated would not be detectable in your survey flights, which is important to consider if you are going to attempt to use “hunting attempts” as a proxy for the distribution of ringed seal lairs. I also wondered whether the survey periods included any snowfall events that would obscure kills & predation attempts, potentially unevenly if part of the study area was surveyed right after a heavy snow. It would also be good to acknowledge temporal variability in seal haul-out behavior (which exists for both seal species). This would influence how many seals were observed and, depending on how your survey effort was distributed over time and space, the location of the hotspots identified in your analysis.

**Response:** See response to R2-31, and the animated gif. We also added a note about possible bias related to snow fall obscuring smaller fox tracks L.483-487 “*The delay between the timing of our flights and the occurrence of fox visits to areas searched by bears may also explain this low overlap. D**ue to constraints related to weather conditions when flying, we may have missed tracks associated with ringed seal structures: snowfall and snow dusting could have obscured Arctic fox tracks, even when larger bear tracks were still visible.*” and signs of hunting success L.196-197 “*(snow fall may sometimes cover blood and small remains obscuring the signs)*”.

A bias on seal haul-out behavior is unlikely, though. The number of seals hauled out does increase from late April to early May, but data are pooled together. Furthermore, the survey period occurs every year at the same time. Therefore, like all other observations, it is representative of the general survey period. We produced results and associated interpretation at coarse spatial and temporal scale, with the intention to take care of most bias. We believe that adding details on topics beyond the scope of this study would risk confusing readers and overcomplicating the manuscript. Given the coarse-scale nature of our data, we aimed to keep the paper concise and focused on the broad spatial associations among four predators and their prey on sea ice.

**Figures**

**R2-37:** For your hotspot and overlap figures, I kept wondering what portions were landfast ice and what portions were pack ice. I realize the extent and positions of these features can change within and between years, but would it make sense to indicate a representative example in Fig 1, e.g. by adding a line for typical landfast ice extent or landfast ice extent from a given year of your study?.

**Response:** We added a dashed line representing the typical extent of landfast ice to Fig.1.

**R2-38:** Figure 2

What does the spatial extent of the grid cells indicate? Is it a minimum convex polygon around all observations of that category? Or what? Please clarify. For Arctic foxes, for example, it looks like the northeast part of the study area wasn’t included in the hotspot analysis and it isn’t clear why. If it’s because there weren’t any fox tracks seen there, then it seems like that should be a cold spot (?).

**Response:** The way the package has trimmed the calculation grids seems to be an artifact of the internal calculation of *sfhotspot* and related to the uneven effort that we had to normalize (and thus the fact that presence and absence are not equal): we did not force a full grid during hotspot calculation to avoid the inclusion of spurious 0 (for non-surveyed cells). The Gi* statistic is a relative measure of value in a cell given all neighboring cells. Therefore a 0 in lightly surveyed area doesn’t carry the same weight like a 0 in a well-surveyed cell. For example, if we have 100 observations in a heavily surveyed cell vs 10 in a lightly surveyed cell, it is easy to standardize based on some effort metric. But a 0 will always be a 0, no matter how we standardize. The method we used is, thus, somewhat similar to “presence only” data in species distribution models. Here, *sfhotspot* created a cell as long as there is at least 1 helicopter point, and will only be able to calculate a cold spot if for a given area there are enough cells with observations around a few surveyed cells with true 0s. However, if it calculates a cold spot, interpretation is challenging: 1) we would need to review how heavily the cells of the cold spots and neighbors were surveyed to make sure it is a true cold spot not a method bias, and 2) at the scale of the study area it would be difficult to properly compare the cold spot to other areas, because we cannot know if the trimmed areas were also cold spots or insignificant. In addition, sfhotspot found some cold spots only for bears at 0.05 < p < 0.1 (p = 0.1 seems lenient given the resolution of our data, we aimed for more conservative parameters).

We removed the cold spot from the figure, because as the reviewer mentioned the figures highlight our main point, and we do not discuss cold spots (or lack thereof). We further mentioned that point directly in the “Hotspot analysis” section to avoid mentioning cold spots altogether for the rest of manuscript.

We re-created Figure 2 in R rather than ArcGIS, because unlike in calculation, we can constrain the hotspot to be on the full grid of the survey area for visualization. Below is the new Fig.2.

**R2-39:** The caption only mentions hotspots, but panel C includes coldspots.

**Response:** As described above (response to R2-38, we removed the cold spot from the figure.

**R2-40:** Silhouettes: Consider making the silhouettes somewhat to scale (e.g., ringed seal smaller than bearded seal). I also didn’t find the silhouettes for panels E and F very intuitive and suggest considering some alternatives. Examples below for kills (ringed or bearded options, with bear eating the seal) and hunting attempts (same as your current panel F silhouette, but species are slightly more to scale and seal is “escaping”).

**Response:** We revised the silhouettes.

**R2-41:** Since these panels have such similar contents, you have an opportunity to reduce visual clutter a bit and make it easier for readers to focus on the actual hotspots. Consider only having the north arrow and scalebar in one of the panels. You could also consider having one legend (potentially off to the side of the figure) instead of having nearly identical legends in all six panels. I also don’t feel that the Hudson Bay label is necessary

**Response:** See figure proposed in Response to R2-38.

**R2-42:** Figure 3 (also largely applicable to Figs S1 and S2)

There’s some untapped potential here, and small changes to your plotting choices would go a long way. Because the hotspots are plotted using outlines instead of filled polygons, it’s hard to see where the actual hotspots are. When there’s a hole in a hotspot, for example, the shading within the hotspot and within the hole are currently the same. Areas of overlap are also very hard to see, especially when the hotspots are complex shapes like in FigS2B. These problems could be resolved by using semitransparent filled polygons for each type of hotspot, resulting in a different/darker color where hotspots overlap. These figures are the key result of your paper so I think it’s important that they be strong, intuitive, easy-to-see visualizations of overlap.

The suggestions about visual clutter in Figure 2 also apply here (e.g., no need to have a north arrow on every single panel). Reducing clutter will make it easier for readers to key in on the message you are trying to convey with the figure.

**Response:** We find the current version identifying overlap with shading easier to read than semitransparent fill. Also, there is no shading in hotspot holes, only the white background. The shading serves only to differentiate areas of overlap from areas exclusive to a given hotspot.

We propose an alternative aesthetic that 1) highlights holes in hotspots with the proposed semi-transparent fill, 2) keeps the overlap area obvious by keeping the shading, which we have made darker (the semi-transparent fill often obscures other features), similar to our original aesthetic.

Note that the Fig. 3K to Fig. 3N (with the structure hotspot) were missing from our original submission. We have added them now for consistency, and referenced them in Results (L.353-355).

**R2-43:** Figures S1 & S2: Year labels like “2019-2022” imply observations from 2019, 2020, 2021, 2022 which is not the case. Perhaps just use a comma instead of a dash.

**Response:** switched for “vs”.

**R2-44:** Figure S2: I suggest doing something more to differentiate the bears vs seals. Could do column-wise (bear panels all in the left column, seals all in the right column), or split into separate figures.

**Response:** Fair point, we have divided them into Fig.S2 (bear-year plots) and Fig.S3 (bear-seal by year plots).

Note that we have also switched the color scheme of Fig.S1 and S2 to the viridis scheme used for the helicopter track animated gif. We had initially kept the color associated to each species in the main text, but they were difficult to distinguish, particularly for the seal plots. The caption is clear enough, and we added our silhouettes again to help identify the purpose of the plots at first glance.

**Tables**

**R2-45:** Table 1: please convert the “Time flown” column from minutes to hours

**Response:** Done. We noted a mistake in this table: some static time was included in the count. We corrected the total amount of hours in the table and the first sentence in Results to report only time moving during the survey.

**R2-46:** I hope these comments are helpful and I wish the authors the best of luck.

**Response:** We believe that feedback from a peer’s outside perspective is always valuable. Thank you again for all the time you devoted to our manuscript, it is greatly appreciated!
